# The CSF1R-Microglia Axis Has Protective Host-Specific Roles During Neurotropic Picornavirus Infection

**DOI:** 10.3389/fimmu.2021.621090

**Published:** 2021-09-09

**Authors:** John Michael S. Sanchez, Ana Beatriz DePaula-Silva, Daniel J. Doty, Tyler J. Hanak, Amanda Truong, Jane E. Libbey, Robert S. Fujinami

**Affiliations:** ^1^Department of Pathology, University of Utah School of Medicine, Salt Lake City, UT, United States; ^2^Department of Oncological Sciences, Huntsman Cancer Institute, Salt Lake City, UT, United States

**Keywords:** microglia, picornavirus, Theiler’s murine encephalomyelitis virus, T cell, innate immunity, virology, demyelination, multiple sclerosis

## Abstract

Viral encephalitis is a major cause of morbidity and mortality, but the manifestation of disease varies greatly between individuals even in response to the same virus. Microglia are professional antigen presenting cells that reside in the central nervous system (CNS) parenchyma that are poised to respond to viral insults. However, the role of microglia in initiating and coordinating the antiviral response is not completely understood. Utilizing Theiler’s murine encephalomyelitis virus (TMEV), a neurotropic picornavirus, and PLX5622, a small molecule inhibitor of colony-stimulating factor 1 receptor (CSF1R) signaling that can deplete microglia in the CNS; we investigated the role of the CSF1R-microglia axis in neurotropic picornavirus infection of C57BL/6J and SJL/J mice. These mouse strains differ in their ability to clear TMEV and exhibit different neurological disease in response to TMEV infection. CSF1R antagonism in C57BL/6J mice, which normally clear TMEV in the CNS, led to acute fatal encephalitis. In contrast, CSF1R antagonism in SJL/J mice, which normally develop a chronic CNS TMEV infection, did not result in acute encephalitis, but exacerbated TMEV-induced demyelination. Immunologically, inhibition of CSF1R in C57BL/6J mice reduced major histocompatibility complex II expression in microglia, decreased the proportion of regulatory T cells in the CNS, and upregulated proinflammatory pathways in CNS T cells. Acute CSF1R inhibition in SJL/J mice had no effect on microglial MHC-II expression and upregulated anti-inflammatory pathways in CNS T cells, however chronic CSF1R inhibition resulted in broad immunosuppression. Our results demonstrate strain-specific effects of the CSF1R-microglia axis in the context of neurotropic viral infection as well as inherent differences in microglial antigen presentation and subsequent T cell crosstalk that contribute to susceptibility to neurotropic picornavirus infection.

## Introduction

In the United States, an estimated 6000 people per year are hospitalized for viral encephalitis at a total cost of up to $540 million annually (1). Vaccination strategies and antiviral agents, including acyclovir and foscarnet, have been efficacious against select neurotropic viruses. However, immunomodulatory agents are also used in the context of acute viral encephalitis, either when no vaccine or antimicrobial drug is available or as an adjunct therapy, with limited benefit (1, 2). There is a clear need to elucidate the necessary components of the antiviral response and the non-essential mediators of immunopathology to develop better immunomodulatory strategies for viral encephalitis patients.

Microglia are myeloid cells that are uniquely found in the central nervous system (CNS) parenchyma and are ideally situated to respond to neurotropic viruses, but the role of microglia in viral encephalitis is not completely understood. Previous work has identified both protective and pathogenic functions of microglia in the antiviral response (3). Indeed, altered expression of genes related to the innate immune response, including pattern recognition receptors and antigen presentation machinery, has been proposed to be an important factor in determining individual susceptibility to viral encephalitis (4, 5). Among the first immune cells to respond to viral insult in the CNS, microglia may be critical determinants for the subsequent steps of the antiviral response.

Theiler’s murine encephalomyelitis virus (TMEV) is a model neurotropic picornavirus that manifests different neurological disease depending on the host strain infected. In C57BL/6J (B6J) mice, TMEV infection results in the development of acute seizures, followed by clearance of the virus within 14 days post infection (DPI), and finally spontaneous recurrent seizures (6). Macrophages rapidly infiltrate the CNS in B6J mice in response to TMEV infection and are pathogenic drivers of the seizure phenotype, but are not necessary for clearance of the virus (7, 8). In contrast, CD4^+^ and CD8^+^ T cells are critical to the TMEV response in the CNS, but are dispensable for the seizure phenotype (8–10). Recently, we and others have shown that microglia, unlike macrophages, are needed for the response against TMEV in B6J mice as microglia-deficient mice succumb to viral encephalitis (7, 11). The mechanisms of microglia-T cell crosstalk in B6J TMEV infection, however, remain poorly understood.

In contrast to B6J mice, SJL/J (SJL) mice challenged with TMEV in the CNS develop acute encephalitis, but are unable to clear the virus and go on to develop TMEV-induced demyelinating disease (TMEV-IDD), a model for multiple sclerosis (6). Microglia and macrophages can display both virus- and self-antigen during the course of disease (12, 13). These myeloid cells can also cause early myelin damage through toxic effectors including proteolytic factors, cytokines and reactive oxygen and nitrogen species (6). Virus-specific CD4^+^ and CD8^+^ T cells contribute to control of the virus, but also drive demyelination through direct cytolytic and cytokine-mediated mechanisms (9, 14). Despite being the more common disease model utilizing TMEV, little is known regarding how microglia coordinate this complex T cell response in the different stages of TMEV-IDD.

To study the role of microglia in the response to TMEV, we deplete microglia using pharmacological inhibition of colony-stimulating factor 1 receptor (CSF1R). Signaling through CSF1R is critical for the survival of myeloid cells (15) and modulation of CSF1R has been shown to have therapeutic benefit in models of neurodegeneration (16) and neuroinflammation (17). PLX5622 is a small molecule inhibitor of CSF1R that has been previously shown to cross the blood-brain barrier to deplete microglia in the CNS (11, 18, 19). This approach is advantageous in that targeting of microglia occurs regardless of host genetic background and can be targeted to specific stages of disease based on when the drug is administered.

Here, we utilize the mouse strain-dependent phenotype of TMEV infection to elucidate the unique and overlapping roles of CSF1R signaling and microglia between closely related hosts. We report that while CSF1R inhibition depletes microglia in both B6J and SJL mice, microglia-deficient B6J mice exhibit aberrancies in the innate and adaptive immune response during acute TMEV infection that are not observed in microglia-deficient SJL mice. In fact, microglia-deficient SJL mice have extended longevity compared to microglia-deficient B6J mice. Still, chronic CSF1R inhibition in SJL mice exacerbates TMEV-IDD − an effect that is partially reversible by discontinuing PLX5622 treatment at the onset of disease and partially recapitulated by initiating PLX5622 treatment at disease onset. Thus, microglia and CSF1R signaling play a protective role in TMEV infection in B6J and SJL mice, but are required at different points during disease depending on the host strain. In B6J mice, microglia and CSF1R signaling are needed for the acute response to TMEV, whereas CSF1R signaling is critically important in the chronic stage of TMEV-IDD in SJL mice.

## Materials and Methods

### Animals

B6J male and SJL female mice were purchased from the Jackson Laboratory (Bar Harbor, ME). The care and use of the mice were performed in accordance with the guidelines prepared by the Committee on Care and Use of Laboratory Animals, Institute of Laboratory Animals Resources, National Research Council.

### PLX5622 Treatment

B6J and SJL mice (4 weeks old) received either AIN-76A Rodent Diet without PLX5622 or AIN-76A Rodent Diet with 1200-mg PLX5622 (free base)/kg (Research Diets, New Brunswick, NJ and Plexxikon, Berkeley, CA) as indicated. Food and water were available *ad libitum*.

### Infection

B6J and SJL mice (5 weeks old) were anesthetized with isoflurane *via* inhalation and infected intracerebrally (i.c.) with 4 × 10^4^ and 3 × 10^5^ plaque forming units (PFUs) of the Daniels (DA) strain of TMEV, respectively. The site of injection was in the post-parietal cortex of the right cerebral hemisphere to a depth of 2 mm. The needle had a William’s collar to limit penetration of the tip to 2 mm. The DA strain was propagated as previously described (20).

### Observations

Mice were observed regularly for weight change, seizure activity, and paralysis. Clinical data was generated using seven to twenty mice per group. Seizure activity was graded using the Racine scale: stage 1, mouth and facial movements; stage 2, head nodding; stage 3, forelimb clonus; stage 4, rearing; stage 5, rearing and falling (11). Clinical score was determined by righting reflex (21). When the proximal end of the mouse’s tail is grasped and twisted to the right and then to the left, a healthy mouse resists being turned over (score of 0). If the mouse is flipped onto its back but immediately rights itself on one side or both sides, it is given a score of 1 or 1.5, respectively. If it rights itself in 1 to 5 s, the score is 2. If righting takes 5 to 10 s, the score is 3. If righting takes 10 to 15 s, the score is 4. If righting takes longer than 15 s, the score is 5.

### Immunohistochemistry

Mice were euthanized at the indicated time points with isoflurane and perfused with phosphate-buffered saline (PBS), followed by a 4% paraformaldehyde phosphate-buffered solution. Brains were cut coronally, and spinal cords were cut transversely and embedded in paraffin by standard methods. Four-μm-thick tissue sections were stained with Luxol fast blue for myelin visualization. TMEV antigen was visualized by the avidin-biotin peroxidase complex technique, using hyperimmune rabbit serum to DA virus (22). Slides were scanned using the Pannoramic MIDI digital slide scanner, visualized using CaseViewer 2.2 digital microscope software, and quantified using QuantCenter image analysis platform (3D HISTECH Ltd., Budapest, Hungary). Six to nine mice per group with at least five sections per mouse were used for quantification. The amount of TMEV antigen was calculated in brain sections by percent positive pixels divided by total non-background pixels. The extent of demyelination was calculated in annotated white matter tracts of the spinal cord by percent of light blue area divided by total tissue area.

### Flow Cytometry

Mice were euthanized at the indicated time points with isoflurane and perfused with PBS. Brain and spinal cord tissues were pooled for each animal, enzymatically dissociated with collagenase (MilliporeSigma, Burlington, MA) and DNase I (Roche, Basel, Switzerland), and subsequently mechanically dissociated by vigorous pipetting. Leukocytes were enriched by Percoll (MilliporeSigma) density gradient centrifugation and counted using the Bio-Rad TC20 automated cell counter (Bio-Rad, Hercules, CA). Cells were treated with Fc blocker (BioLegend, San Diego, CA), stained with the indicated anti-mouse antibodies for 30 min at 4°C [V500 anti-mouse CD45 (BD Bioscience, San Jose, CA), APC anti-mouse CD11b (eBioscience, San Diego, CA), PE anti-mouse major histocompatibility complex class II (MHC-II) (eBioscience), BV421 anti-mouse CD3 (eBioscience), APC anti-mouse CD4 (eBioscience), FITC anti-mouse CD8 (eBioscience), BV711 anti-mouse CD25 (eBioscience), and PE anti-mouse FoxP3 (eBioscience)], and analyzed by flow cytometry. CNS-derived cells were stained and analyzed individually for each mouse. Live cells were determined by forward and side scatter fluorescence and viability dye [Zombie Violet (BioLegend) or Live/Dead Aqua (ThermoFisher, Waltham, MA)] on a BD LSRFortessa X-20 Cell Analyzer (BD Bioscience). Tissue from four to seven mice per group was used to generate flow cytometry data. Flow cytometry data analysis was performed using FlowJo software (FlowJo, Ashland, OR).

### Plaque Assay

Mice were euthanized at the indicated time points with isoflurane and perfused with PBS. Five mice per group were used to generate plaque assay data. Brains were harvested, snap frozen in liquid nitrogen, and stored at -80°C until use. Each brain was weighed and homogenized in 0.5 ml PBS. The homogenate was centrifuged and the supernatant was examined for the presence of infectious virus *via* plaque assay on BHK-21 cells as previously described (20).

### RNA Isolation and Library Preparation for RNA Sequencing

Brains were harvested and immune cells were isolated as described above. Ten mice per group were used for cell isolation. CD4^+^ and CD8^+^ T cells were enriched using the negative selection Easy Sep Mouse CD4+ T cell Isolation Kit and Easy Sep Mouse CD8+ T cell Isolation Kit (STEMCELL Technologies, Vancouver, Canada). RNA was immediately isolated using RNeasy (QIAGEN), according to the manufacturer’s protocol, and stored at -80°C. Illumina TruSeq stranded RNA Kit with Ribo-Zero Gold (Illumina, San Diego CA) was used to prepare cDNA libraries for RNA-Seq according to the manufacturer’s protocol. HiSeq 50 cycle single-read sequencing was performed on an Illumina HiSeq 2500 instrument. RNA-Seq and the bioinformatic analyses of the results obtained from the RNA-Seq experiment were done by the High Throughput Genomics Core Facility in the Huntsman Cancer Institute at the University of Utah.

### Statistics

GraphPad Prism 7 (GraphPad Software, San Diego, CA) was used for statistical analyses. To compare two groups, a Student *t* test was performed. To compare more than two groups, a one-way ANOVA was performed. To compare survival between groups a log-rank test was performed. Weight loss and clinical score were compared by two-way ANOVA. P < 0.05 was considered statistically significant.

## Results

### Acute Inhibition of CSF1R Signaling Using PLX5622 Depletes Microglia in the CNS, but Does Not Affect Recruitment of Leukocytes in Response to I.c TMEV Infection

**Figure 1:**
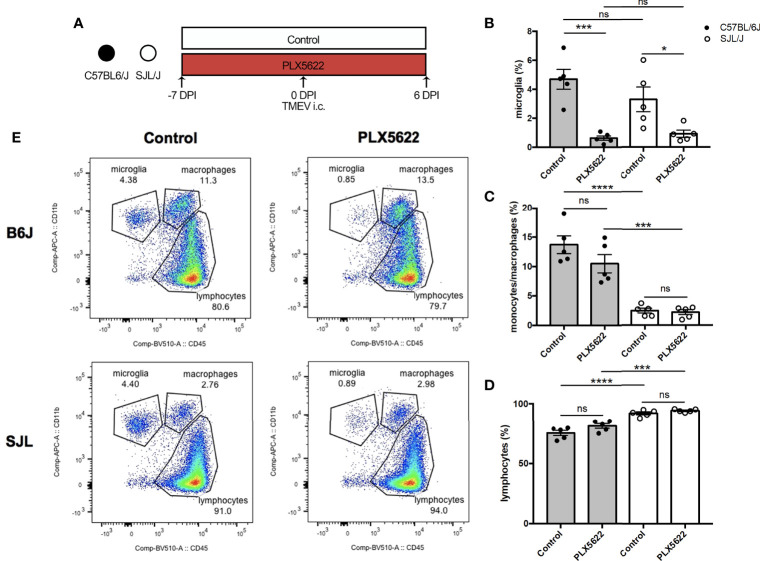
Short-term PLX5622 treatment does not impede TMEV-induced recruitment of leukocytes into the CNS. **(A)** C57BL/6J mice and SJL/J mice were treated with a PLX5622-supplemented or control diet seven days prior to i.c. TMEV infection and sacrificed at 6 DPI for analysis (n = 5 mice per group). Proportion of **(B)** CD45^lo^CD11b^+^ microglia, **(C)** CD45^hi^CD11b^+^ monocytes/macrophages, and **(D)** of CD45^+^CD11b^-^ lymphocytes from the CNS (combined brain and spinal cord). **(E)** Representative flow plots demonstrating the gating strategy for CNS leukocytes. Data are presented as mean ± SEM. *P ≤ 0.05, ***P ≤ 0.001, ****P ≤ 0.0001, ns, not significant; one-way ANOVA for **(B–D)**.

We have previously shown that depletion of microglia in B6J mice using PLX5622 results in fatal viral encephalitis when challenged with i.c. TMEV infection, even with as little as 40 PFUs of TMEV (11). Based on this previous observation, we first sought to investigate whether CSF1R-mediated depletion of microglia in B6J and SJL mice would affect the acute infiltration of peripheral immune cells into the CNS in response to TMEV infection. To do this, we fed mice a diet supplemented with PLX5622 or the same diet formulation without PLX5622 starting one week prior to i.c. infection with TMEV (**Figure 1A**). We subsequently analyzed CNS (combined brain and spinal cord) leukocytes by flow cytometry at 6 DPI, a time point preceding mortality in microglia-depleted B6J mice (11). Microglia (CD45^lo^CD11b^+^) were significantly depleted in PLX5622-treated animals, regardless of genetic background (**Figure 1B**). As previously described, microglia-competent B6J mice have a higher proportion of CNS monocytes/macrophages and a lower proportion of lymphocytes compared to microglia-competent SJL mice (**Figures 1C, D**) (23). Importantly, CNS monocytes/macrophages (CD45^hi^CD11b^+^) and lymphocytes (CD45^hi^CD11b^-^) were not impeded by PLX5622 treatment in both B6J and SJL mice (**Figures 1B–D**). This suggests that microglia are not necessary for acute recruitment of peripheral immune cells into the CNS upon TMEV challenge and is in agreement with previous studies of other neurotropic viruses (19, 24, 25).

### CSF1R-Mediated Depletion of Microglia Hinders Acute Antiviral Response to TMEV in B6J Mice, but Not SJL Mice

**Figure 2:**
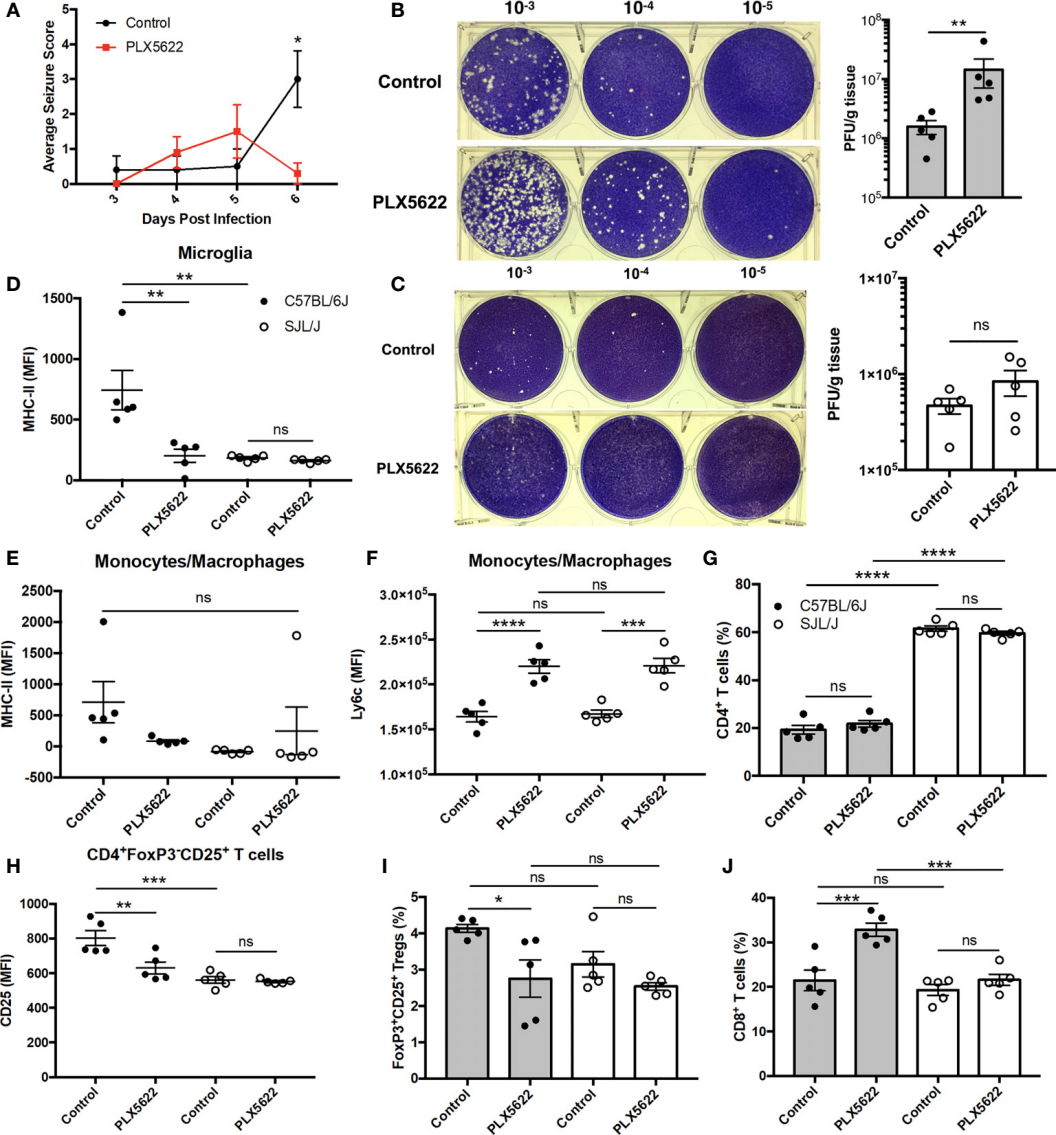
The contribution of microglia to acute control of TMEV CNS infection depends on host genetic background. **(A)** Seizure severity over time in C57BL/6J mice treated with PLX5622 seven days prior to i.c. TMEV infection (n = 10 mice per group). Representative images and quantification of plaque assays from the brains of TMEV-infected **(B)** C57BL/6J mice and **(C)** SJL/J mice. MHC-II expression in CNS **(D)** microglia and **(E)** monocytes/macrophages. **(F)** Ly6c expression in CNS monocytes/macrophages. **(G)** Proportion of CNS CD4^+^ T cells. **(H)** CD25 expression in CNS CD4^+^FoxP3^-^CD25^+^ T cells. **(I)** Proportion of CD4^+^FoxP3^+^CD25^+^ Tregs of CNS CD4^+^ T cells. **(J)** Proportion of CD8^+^ T cells of CNS CD3^+^ T cells. Data are presented as mean ± SEM with 5 mice per group for **(B–J)**. *P ≤ 0.05, **P ≤ 0.01, ***P ≤ 0.001, ****P ≤ 0.0001, ns, not significant; Mann-Whitney U for **(A)**, Student t test for **(B, C)**, and one-way ANOVA for **(D–J)**.

Given that acute PLX5622 treatment specifically depleted microglia in the CNS, while CNS-infiltrating monocytes/macrophages and lymphocytes remained intact, we investigated the role of microglia in TMEV infection. We observed a decrease in seizure severity in microglia-deficient B6J mice at 6 DPI (**Figure 2A**), but not in overall seizure incidence (data not shown), that is confounded by the onset of paralysis that we and others have previously described (11, 25). In addition, we did not observe seizures in SJL mice regardless of PLX5622 treatment (data not shown). We performed plaque assays using lysate from the brains of TMEV-infected mice at 6 DPI. We found that microglia-deficient B6J mice exhibited higher titers of TMEV compared to microglia-competent B6J mice (**Figure 2B**). Surprisingly, at this time point the viral titers between microglia-deficient and microglia-competent SJL mice were not significantly different (**Figure 2C**). The relative importance of microglia in the acute antiviral response to TMEV between mouse strains was also reflected in flow cytometric analysis of CNS leukocytes. First comparing the antigen presentation capability of microglia between microglia-competent B6J and SJL mice, we found that B6J microglia have higher MHC-II expression in response to TMEV compared to SJL mice (**Figure 2D**). We then looked at the antigen presentation capability in the remaining microglia of PLX5622-treated animals and observed lower MHC-II expression in the remaining microglia (**Figure 2D**) of PLX5622-treated B6J mice compared to control-treated B6J mice. However, MHC-II was unaffected by PLX5622 treatment in infiltrating monocytes/macrophages of B6J mice (**Figure 2E**). Nor was MHC-II expression affected by PLX5622 treatment in microglia and infiltrating monocyte/macrophage populations of SJL mice (**Figures 2D, E**). In contrast, PLX5622 treatment increased Ly6c expression in the monocyte/macrophage compartment in both B6J and SJL mice (**Figure 2F**), suggesting the relative immaturity and/or a proinflammatory phenotype of these cells, as previously described (19, 26). Therefore, microglial expression of MHC-II in response to TMEV infection is a key difference between B6J and SJL mice at baseline and is specifically downregulated in microglia-depleted B6J mice.

Because of the deficiencies we observed in the innate immune response of microglia-deficient B6J mice, we subsequently looked at the adaptive immune response. In agreement with previous studies (27), we observed a greater proportion of helper CD4^+^ T cells, which are necessary for activation of other lymphocytes, in the CNS of SJL mice compared to B6J mice (**Figure 2G**). However, there was no difference in the proportion of CNS CD4^+^ T cells within mouse strains with PLX5622 treatment (**Figure 2G**). We ascertained that helper CD4^+^ T cells were less activated in microglia-competent SJL mice compared to microglia-competent B6J mice, as evidenced by lower CD25 expression (**Figure 2H**). Additionally, we measured that CD4^+^ T cells were less activated in microglia-deficient B6J mice compared to microglia-competent B6J mice (**Figure 2H**). Importantly, our analysis of CD25 expression excluded CD4^+^FoxP3^+^ T cells as regulatory T cells (Tregs) (CD4^+^CD25^+^FoxP3^+^ T cells) were also decreased in microglia-depleted B6J mice (**Figure 2I**). In addition to the altered helper T cell response, we found that cytotoxic CD8^+^ T cells, were increased in microglia-deficient B6J mice (**Figure 2J**). Interestingly we did not see similar alterations in the T cell response in microglia-deficient SJL mice compared to microglia-competent SJL mice (**Figures 2H–J**). Together, this suggests that CSF1R-mediated microglia depletion selectively impairs the innate and adaptive immune response of B6J mice at this acute timepoint while antiviral immunity in microglia-deficient SJL mice is grossly spared.

### SJL Mice Undergoing Chronic CSF1R Inhibition Do Not Succumb to Acute Viral Encephalitis, but Exhibit Exacerbated TMEV-IDD

**Figure 3:**
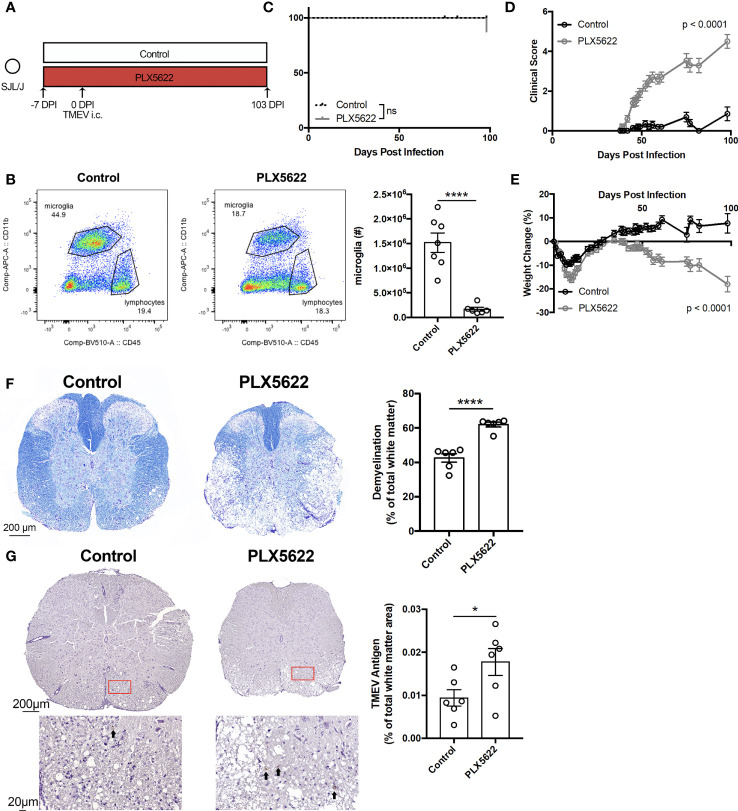
SJL/J mice chronically treated with PLX5622 exhibit exacerbated TMEV-induced demyelinating disease. **(A)** SJL/J mice were treated with a PLX5622-supplemented or control diet seven days prior to i.c. TMEV infection and sacrificed at 103 DPI for analysis. **(B)** Representative flow plots and quantification of CNS microglia (n = at least 6 mice per group). **(C)** Survival, **(D)** clinical score, and **(E)** weight change corresponding to **(A)** (n = 20 mice per group). **(F)** Representative luxol fast blue staining and **(G)** TMEV antigen staining (arrows highlight positive staining) in thoracic spinal cord cross sections and quantification (n = 6 mice per group, at least 5 sections per mouse). Data are presented as mean ± SEM. *P ≤ 0.05, ****P ≤ 0.0001, ns, not significant; Student *t* test for **(B, F, G)**, log-rank test for **(C)**, and two-way ANOVA for **(D, E)**.

To investigate the effect of long-term CSF1R inhibition on TMEV-IDD, we infected SJL mice one week after initiating PLX5622 (**Figure 3A**). We observe that microglia (CD45^lo^CD11b^+^) remain suppressed in PLX5622-treated animals at 103 DPI (**Figure 3B**). Surprisingly, unlike microglia-deficient B6J mice challenged with TMEV that uniformly succumb to viral encephalitis within 10 DPI (11), microglia-deficient, TMEV-infected SJL mice survive well past that time point (**Figure 3C**). Still, chronic CSF1R inhibition in SJL mice results in more severe symptoms of TMEV-IDD, as evidenced by clinical score and weight loss, compared to control SJL mice (**Figures 3D, E**). Histological analyses also revealed a greater extent of demyelination and TMEV antigen in the spinal cords of PLX5622-treated SJL mice (**Figures 3F, G**). These data suggest that CSF1R signaling, although not necessary for the acute TMEV response in SJL mice, plays a critical role in sustained infection by limiting TMEV-IDD.

### Long-Term PLX5622 Treatment Results in Altered T Cell Response and Systemic Immunosuppression

**Figure 4:**
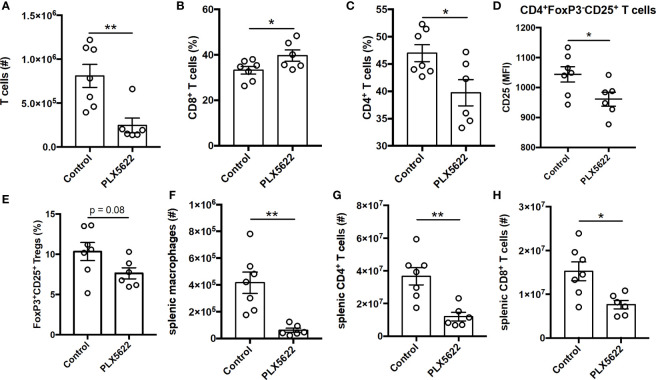
Long term PLX5622 supplementation in chronically infected SJL/J mice alters CNS T cell response and has systemic effects. SJL/J mice were treated with a PLX5622-supplemented or control diet seven days prior to i.c. TMEV infection and sacrificed at 103 DPI for analysis (n = at least 6 mice per group). **(A)** Number of CNS T cells. Proportion of **(B)** CD8^+^ T cells and **(C)** CD4^+^ T cells of CNS CD3^+^ T cells. **(D)** CD25 expression in CNS CD4^+^FoxP3^-^CD25^+^ T cells. **(E)** Proportion of CD4^+^FoxP3^+^CD25^+^ Tregs of CNS CD4^+^ T cells. Number of **(F)** splenic CD11b^+^F4/80^+^ macrophages, **(G)** splenic CD4^+^ T cells, and **(H)** splenic CD8^+^ T cells. Data are presented as mean ± SEM. *P ≤ 0.05, **P ≤ 0.01; Student *t* test for **(A–H)**.

TMEV-specific CD4^+^ and CD8^+^ T cells are necessary for control of virus replication, but are not required for TMEV-IDD (9). Indeed, unlike short-term PLX5622 treatment, long-term PLX5622 treatment of SJL mice resulted in a decrease in the absolute number of CNS-infiltrating T cells (**Figure 4A**). Among the CNS-infiltrating T cells, we observe an increase in the proportion of CD8^+^ T cells and a corresponding decrease in the proportion of CD4^+^ T cells (**Figures 4B, C**). Long-term PLX5622 treatment is also associated with less activation of CNS CD4^+^ T cells, as measured using CD25 expression (**Figure 4D**), and a trending decrease in CNS Tregs (**Figure 4E**). Outside of the CNS, SJL mice on long-term PLX5622 exhibit fewer splenic macrophages, splenic CD4^+^ T cells, and splenic CD8^+^ T cells (**Figures 4F–H**). Our findings agree with recent reports suggesting chronic CSF1R inhibition affects other immune cells beyond the CNS microglia compartment (28, 29). Together, these data suggest that sustained CSF1R signaling is required for the accumulation of CNS T cells and maintaining T cell activation during chronic TMEV infection.

### Immune Reconstitution by Discontinuing PLX5622 Treatment Ameliorates Select Measures of TMEV-IDD

**Figure 5:**
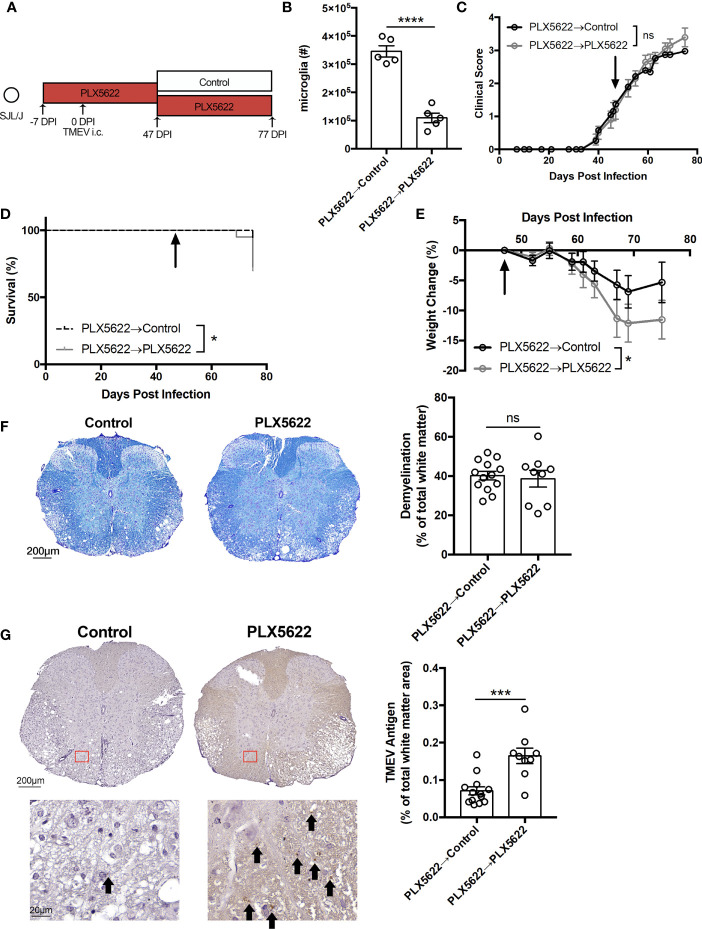
Cessation of PLX5622 suppresses TMEV-associated pathology in microglia-deficient SJL/J mice. **(A)** SJL/J mice were treated with a PLX5622-supplemented diet seven days prior to i.c. TMEV infection, switched to a control diet or remained on the PLX5622-supplemented diet at 47 DPI, and were sacrificed at 77 DPI for analysis. **(B)** Number of microglia (n = 5 mice per group). **(C)** Clinical score, **(D)** survival, and **(E)** weight change corresponding to **(A)** (n = 20 mice per group). **(F)** Representative luxol fast blue staining and **(G)** TMEV antigen staining (arrows highlight positive staining) in thoracic spinal cord cross sections and quantification (n = at least 9 mice per group, at least 5 sections per mouse). Data are presented as mean ± SEM. *P ≤ 0.05, ***P ≤ 0.001, ****P ≤ 0.0001, ns, not significant; Student *t* test for **(B, F, G)**, log-rank test for **(D)**, and two-way ANOVA for **(C, E)**.

In order to test whether we could rescue the phenotype of SJL mice treated with long-term PLX5622, half of the cohort of SJL mice on a PLX5622-supplemented diet were switched to the control diet at 47 DPI, corresponding to the onset of clinical signs of TMEV-IDD (**Figure 5A**). Thirty days post-PLX5622 discontinuation, we observed a significant repopulation of the CNS microglia niche (**Figure 5B**), which has previously been shown to be achieved *via* both CNS-resident microglia progenitors (15) and peripheral monocytes (30). While clinical score was not significantly altered by switching SJL mice away from the PLX5622-containing diet (**Figure 5C**), mortality and weight loss were decreased with cessation of PLX5622 in SJL mice (**Figures 5D, E**). Histologically, we observed no difference in spinal cord demyelination between microglia-deficient and microglia-repopulated mice (**Figure 5F**), but TMEV antigen in the spinal cord with discontinuation of PLX5622 (**Figure 5G**).

**Figure 6:**
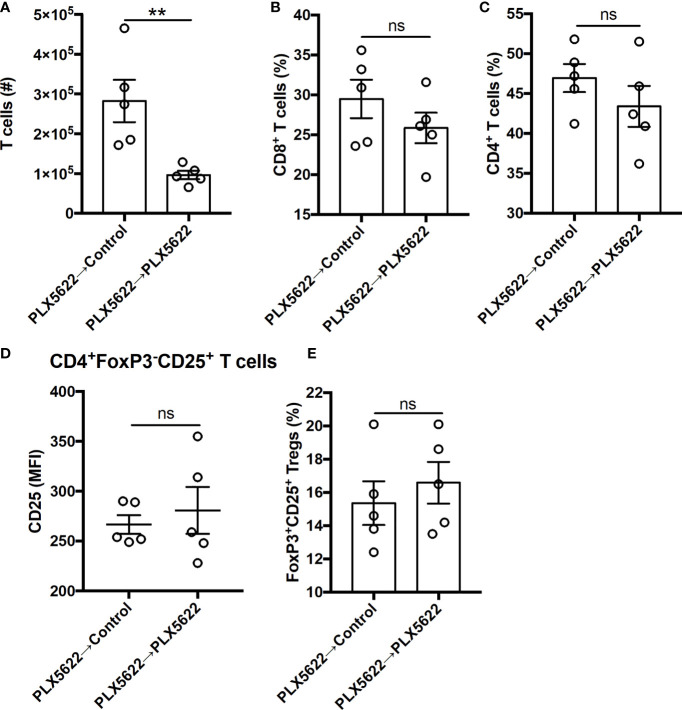
Deficits in CNS T cell infiltration in chronically infected, microglia-deficient SJL/J mice can be reversed by discontinuing PLX5622. SJL/J mice were treated with a PLX5622-supplemented diet seven days prior to i.c. TMEV infection, switched to a control diet or remained on the PLX5622-supplemented diet at 47 DPI, and were sacrificed at 77 DPI for analysis (n = 5 mice per group). **(A)** Number of CNS T cells. Proportion of **(B)** CD8^+^ T cells and **(C)** CD4^+^ T cells of CNS CD3^+^ T cells. CD25 expression in CNS CD4^+^FoxP3^-^CD25^+^ T cells. **(E)** Proportion of CD4^+^FoxP3^+^CD25^+^ Tregs of CNS CD4^+^ T cells. Data are presented as mean ± SEM. **P ≤ 0.01, ns, not significant; Student *t* test for **(A–E)**.

In addition to the changes in innate immunity, the number of CNS-infiltrating T cells was increased with cessation of PLX5622 in SJL mice (**Figure 6A**). However, among the CNS-infiltrating T cells, no difference in the proportion of CD8^+^ T cells and CD4^+^ T cells (**Figures 6B, C**) was noted. We also did not see any differences in the activation of CD4^+^ T cells nor the proportion of Tregs (**Figures 6D, E**). Thus, exacerbated TMEV-IDD due to long-term CSF1R inhibition is partially reversible by discontinuing PLX5622 and indicates that the microglia-T cell axis plays a protective role during sustained TMEV infection at disease onset.

### Depleting Microglia During Sustained TMEV Infection Exacerbates Symptoms of TMEV-IDD Without Hindering T Cell Infiltration Into the CNS

**Figure 7:**
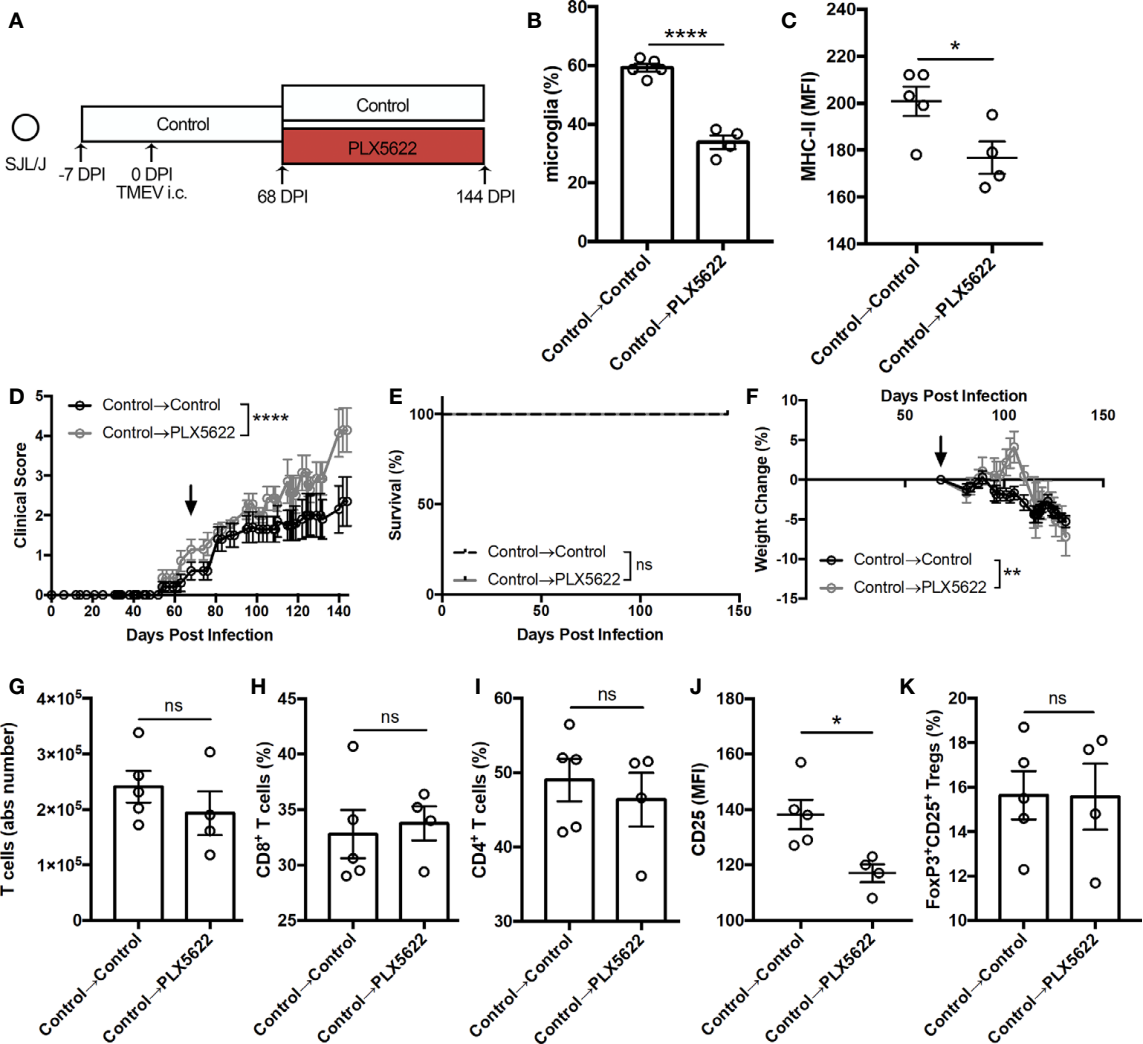
Initiating PLX5622 during chronic TMEV infection exacerbates clinical score while maintaining T cell infiltration. **(A)** SJL/J mice were treated with a control diet seven days prior to i.c. TMEV infection, switched to a PLX5622-supplemented diet or remained on the control diet at 68 DPI, and were sacrificed at 144 DPI for analysis. **(B)** Proportion and **(C)** MHC-II expression of microglia. **(D)** Clinical score, **(E)** survival, and **(F)** weight change corresponding to **(A)** (n = 7-10 mice per group). **(G)** Number of CNS T cells. Proportion of **(H)** CD8^+^ T cells and **(I)** CD4^+^ T cells of CNS CD3^+^ T cells. **(J)** CD25 expression in CNS CD4^+^FoxP3^-^ T cells. **(K)** Proportion of CD4^+^FoxP3^+^CD25^+^ Tregs of CNS CD4^+^ T cells. Data are presented as mean ± SEM with 4-5 mice per group for **(B)**.**(C, G–K)**. *P ≤ 0.05, **P ≤ 0.01; ****P ≤ 0.0001, ns, not significant. Student *t* test for **(B, C**, **G–K)**, log-rank test for **(E)**, and two-way ANOVA for **(D, F)**.

We also investigated whether CSF1R signaling was necessary for the antiviral response during sustained TMEV infection by initiating PLX5622 treatment at the chronic stage of infection. Starting SJL mice on a control diet, we switched half the cohort to a PLX5622-supplemented diet at the onset of disease (68 DPI) while keeping half the cohort on the control diet (**Figure 7A**). At 144 DPI, we observed a significant reduction in the proportion of CNS microglia (**Figure 7B**) in SJL mice switched to the PLX5622-supplemented diet as well as a reduction in microglia expression of MHC-II (**Figure 7C**). Interestingly, we noted exacerbated paralysis in SJL mice depleted of microglia at this chronic time point (**Figure 7D**), but observed no difference in mortality (**Figure 7E**) and decreased weight loss (**Figure 7F**) in microglia-deficient mice compared to microglia-competent mice. In the T cell compartment, we measured similar numbers of CNS T cells (**Figure 7G**) and similar proportions of CD8^+^ and CD4^+^ T cells (**Figures 7H, I**). However, we observed lower activation as measured by CD25 expression in CD4^+^ T cells (**Figure 7J**) and comparable proportions of Tregs (**Figure 7K**) in microglia-deficient mice compared to microglia-competent mice. These data support that CSF1R signaling is necessary after the onset of TMEV-IDD for microglia-mediated stimulation of helper T cells and an effective antiviral response.

### Acute Depletion of Microglia in TMEV-Infected B6J and SJL Mice Results in Altered CNS CD4^+^ and CD8^+^ T Cell Transcriptomes

**Figure 8:**
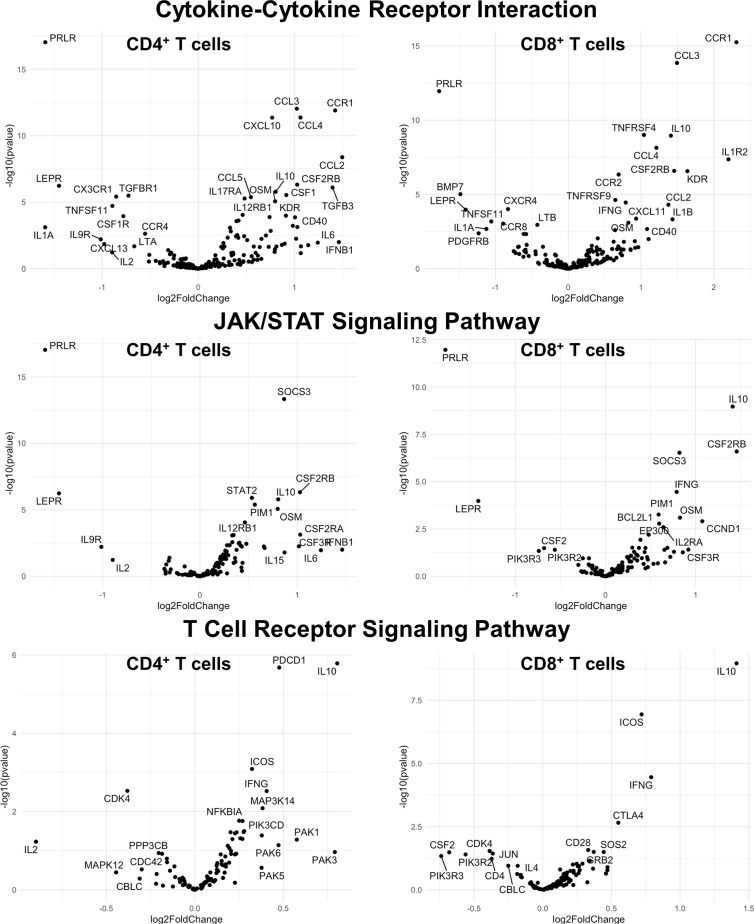
Genes related to cytokine-cytokine receptor interaction, JAK/STAT signaling, and T cell receptor signaling are enriched in CD4^+^ and CD8^+^ T cells from the CNS of microglia-deficient B6J mice compared to microglia-competent B6J mice. C57BL/6J mice were treated with a PLX5622-supplemented or control diet seven days prior to i.c. TMEV infection and sacrificed at 6 DPI for analysis (n = 10 mice per group). Volcano plots of RNA-seq transcriptome data displaying gene expression within the indicated KEGG pathways of enriched CD4^+^ and CD8^+^ T cells from the CNS of microglia-deficient C57BL/6J mice relative to microglia-competent C57BL/6J mice.

**Figure 9:**
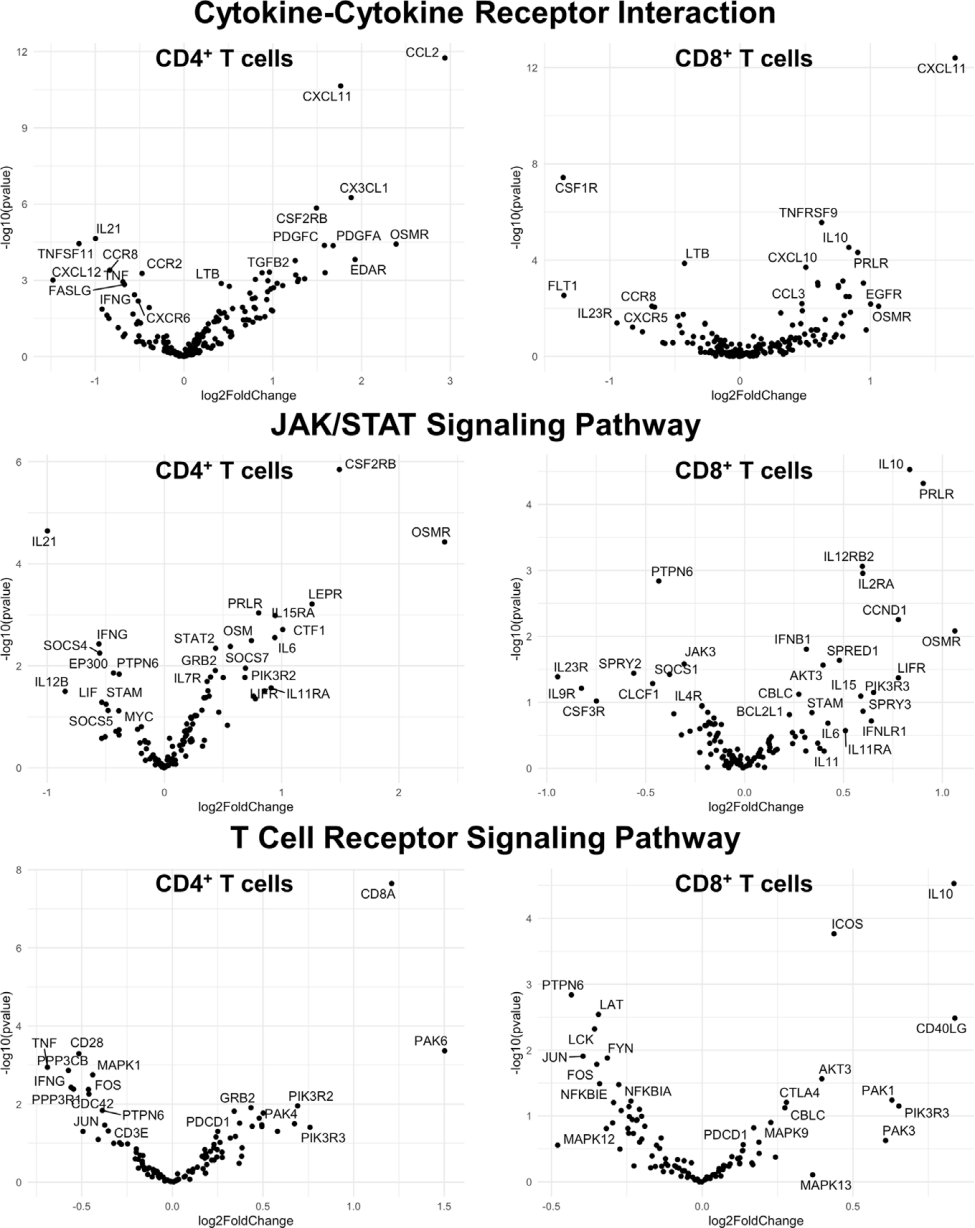
Genes related to cytokine-cytokine receptor interaction, JAK/STAT signaling, and T cell receptor signaling are differentially expressed in CD4^+^ and CD8^+^ T cells from the CNS of microglia-deficient SJL mice compared to microglia-competent SJL mice. SJL/J mice were treated with a PLX5622-supplemented or control diet seven days prior to i.c. TMEV infection and sacrificed at 6 DPI for analysis (n = 10 mice per group). Volcano plots of RNA-seq transcriptome data displaying gene expression within the indicated KEGG pathways of enriched CD4^+^ and CD8^+^ T cells from the CNS of microglia-deficient SJL/J mice relative to microglia-competent SJL/J mice.

Finally, we hypothesized that CSF1R-mediated depletion of microglia in B6J and SJL mice would result in altered function of CD4^+^ and CD8^+^ T cells. To test this hypothesis, we performed RNA-Seq on enriched populations of CNS CD4^+^ T cells and CD8^+^ T cells from B6J and SJL mice treated with PLX5622 or control diet at 6 DPI (**Figure 1A**). This acute time was chosen as CNS immune cell infiltration was not affected in these mouse strains (**Figures 1C, D**), while the ability of these strains to control virus replication was significantly different (**Figures 2B, C**). As a caveat to our RNA-Seq data, we performed negative selection to isolate CD4^+^ and CD8^+^ T cells from the CNS to minimize CD3, CD4, and CD8 binding and subsequent changes in transcriptome associated with positive selection, but the negative selection kits used were not specific for CNS leukocyte isolation. Comparing microglia-competent and microglia-deficient TMEV-infected B6J mice, we find that genes related to JAK/STAT signaling, cytokine signaling, and T cell receptor signaling are highly upregulated in CNS CD4^+^ and CD8^+^ T cells of microglia-deficient B6J mice (**Figure 8** and **Supplemental Tables 1–6**; lists of individual genes used for pathway analysis can be found in the Supplementary Materials). In contrast, comparing microglia-competent and microglia-deficient TMEV-infected SJL mice reveals relatively fewer alterations in these pathways (**Figure 9** and **Supplemental Tables 7–12**). In terms of specific differentially expressed genes, *Ifng* and *Il10* are enriched in both CD4^+^ and CD8^+^ T cells from the CNS of microglia-deficient B6J mice compared to microglia-competent B6J mice, whereas *Ifng* is downregulated in CD4^+^ T cells and *Il10* is upregulated in CD8^+^ T cells in the CNS of microglia-deficient SJL mice compared to microglia-competent SJL mice. These pathway analyses within mouse strains demonstrate a deregulated, overactive inflammatory response in the context of microglia depletion in B6J mice. In contrast, microglia depletion in SJL mice produce a muted antiviral response at this acute timepoint.

**Figure 10:**
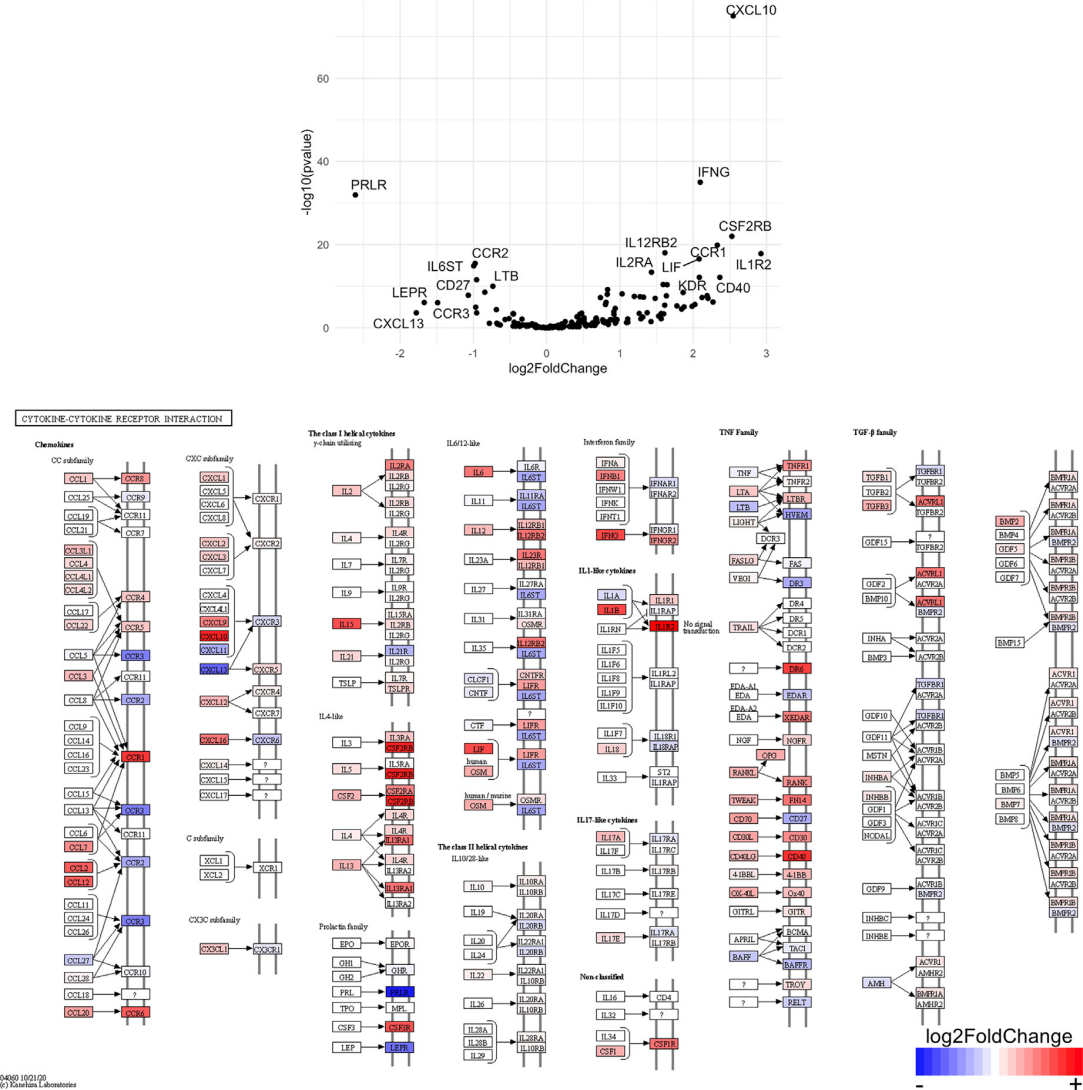
Genes related to cytokine-cytokine receptor interaction are enriched in CD4^+^ T cells from the CNS of microglia-deficient B6J mice compared to microglia-deficient SJL mice. C57BL/6J and SJL/J mice were treated with a PLX5622-supplemented diet seven days prior to i.c. TMEV infection and sacrificed at 6 DPI for analysis (n = 10 mice per group). Volcano plot of RNA-seq transcriptome data and KEGG map display cytokine-cytokine receptor interaction-related gene expression in enriched CD4^+^ T cells from the CNS of microglia-deficient C57BL/6J mice relative to microglia-deficient SJL/J mice.

**Figure 11:**
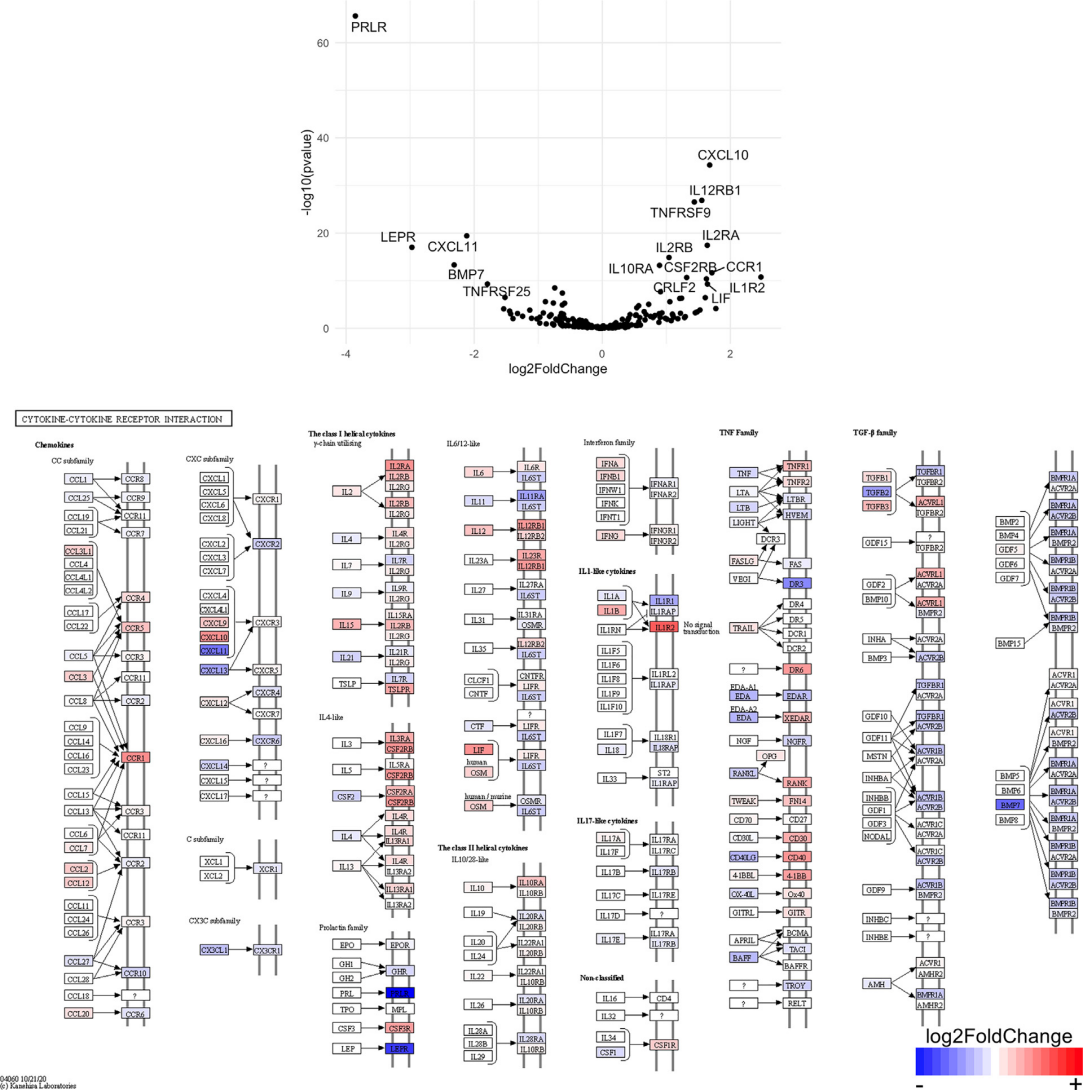
Genes related to cytokine-cytokine receptor interaction are enriched in CD8^+^ T cells from the CNS of microglia-deficient B6J mice compared to microglia-deficient SJL mice. C57BL/6J and SJL/J mice were treated with a PLX5622-supplemented diet seven days prior to i.c. TMEV infection and sacrificed at 6 DPI for analysis (n = 10 mice per group). Volcano plot of RNA-seq transcriptome data and KEGG map display cytokine-cytokine receptor interaction-related gene expression in enriched CD8^+^ T cells from the CNS of microglia-deficient C57BL/6J mice relative to microglia-deficient SJL/J mice.

**Figure 12:**
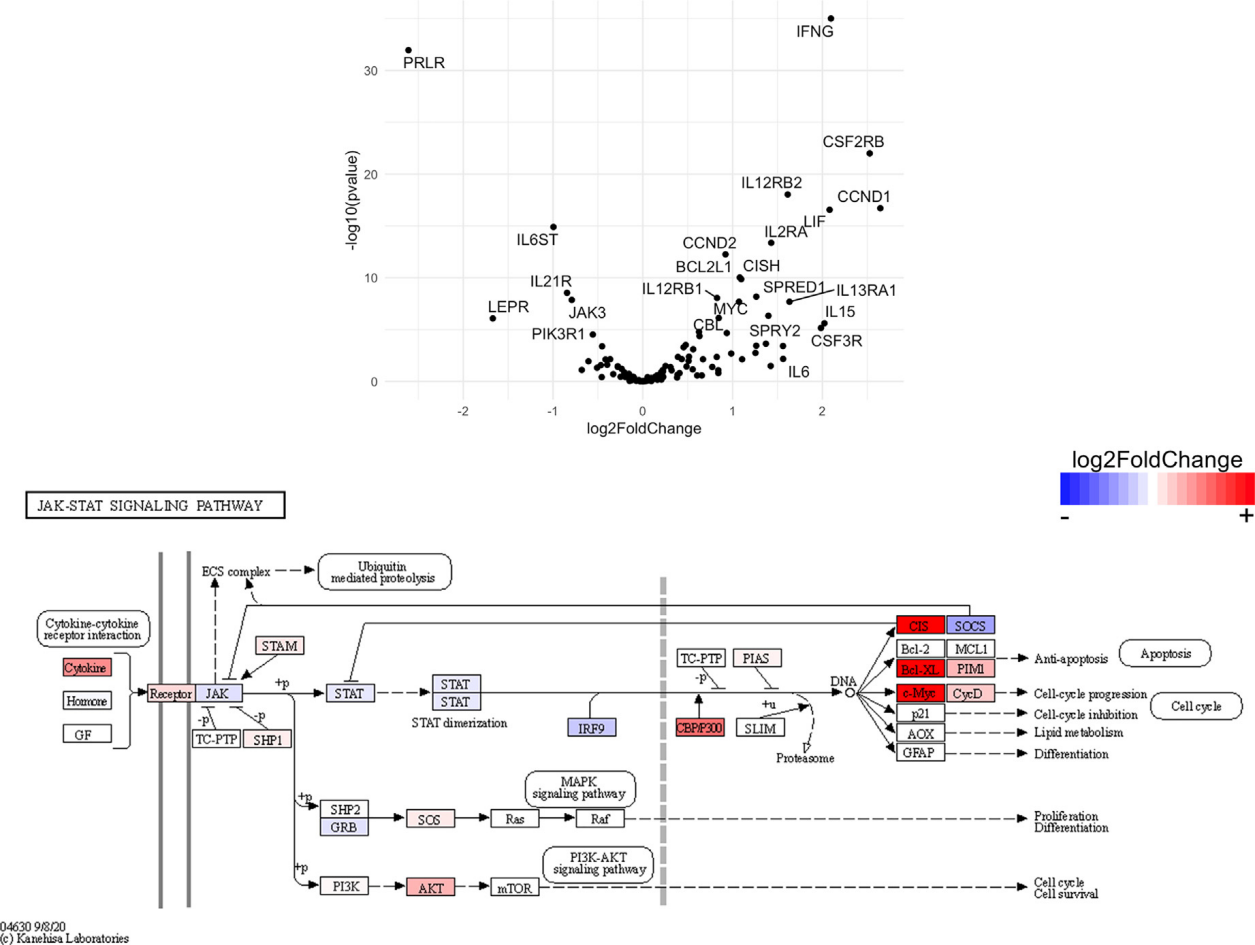
Genes related to JAK/STAT signaling are enriched in CD4^+^ T cells from the CNS of microglia-deficient B6J mice compared to microglia-deficient SJL mice. C57BL/6J and SJL/J mice were treated with a PLX5622-supplemented diet seven days prior to i.c. TMEV infection and sacrificed at 6 DPI for analysis (n = 10 mice per group). Volcano plot of RNA-seq transcriptome data and KEGG map display JAK/STAT signaling-related gene expression in enriched CD4^+^ T cells from the CNS of microglia-deficient C57BL/6J mice relative to microglia-deficient SJL/J mice.

**Figure 13:**
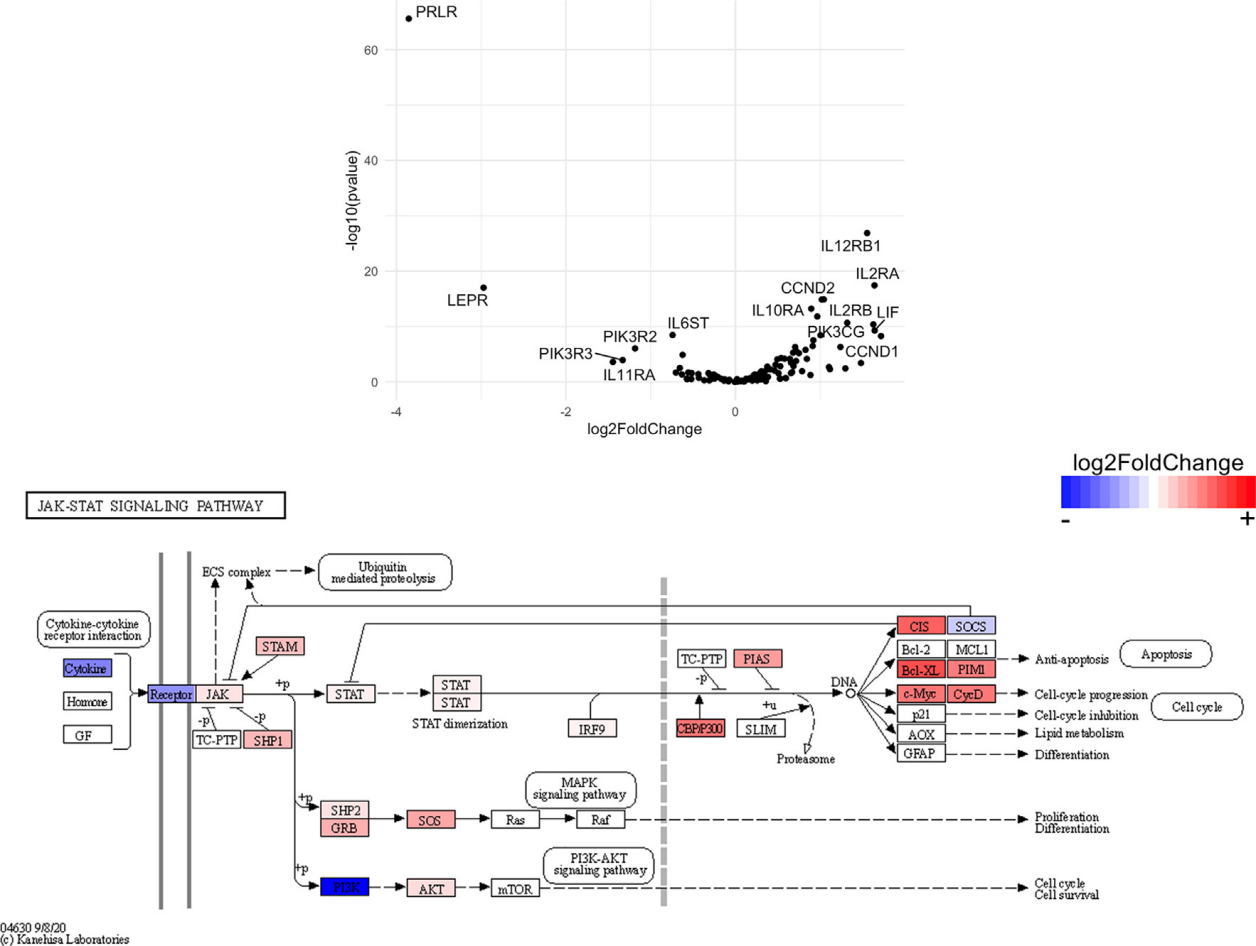
Genes related to JAK/STAT signaling are enriched in CD8^+^ T cells from the CNS of microglia-deficient B6J mice compared to microglia-deficient SJL mice. C57BL/6J and SJL/J mice were treated with a PLX5622-supplemented diet seven days prior to i.c. TMEV infection and sacrificed at 6 DPI for analysis (n = 10 mice per group). Volcano plot of RNA-seq transcriptome data and KEGG map display JAK/STAT signaling-related gene expression in enriched CD8^+^ T cells from the CNS of microglia-deficient C57BL/6J mice relative to microglia-deficient SJL/J mice.

**Figure 14:**
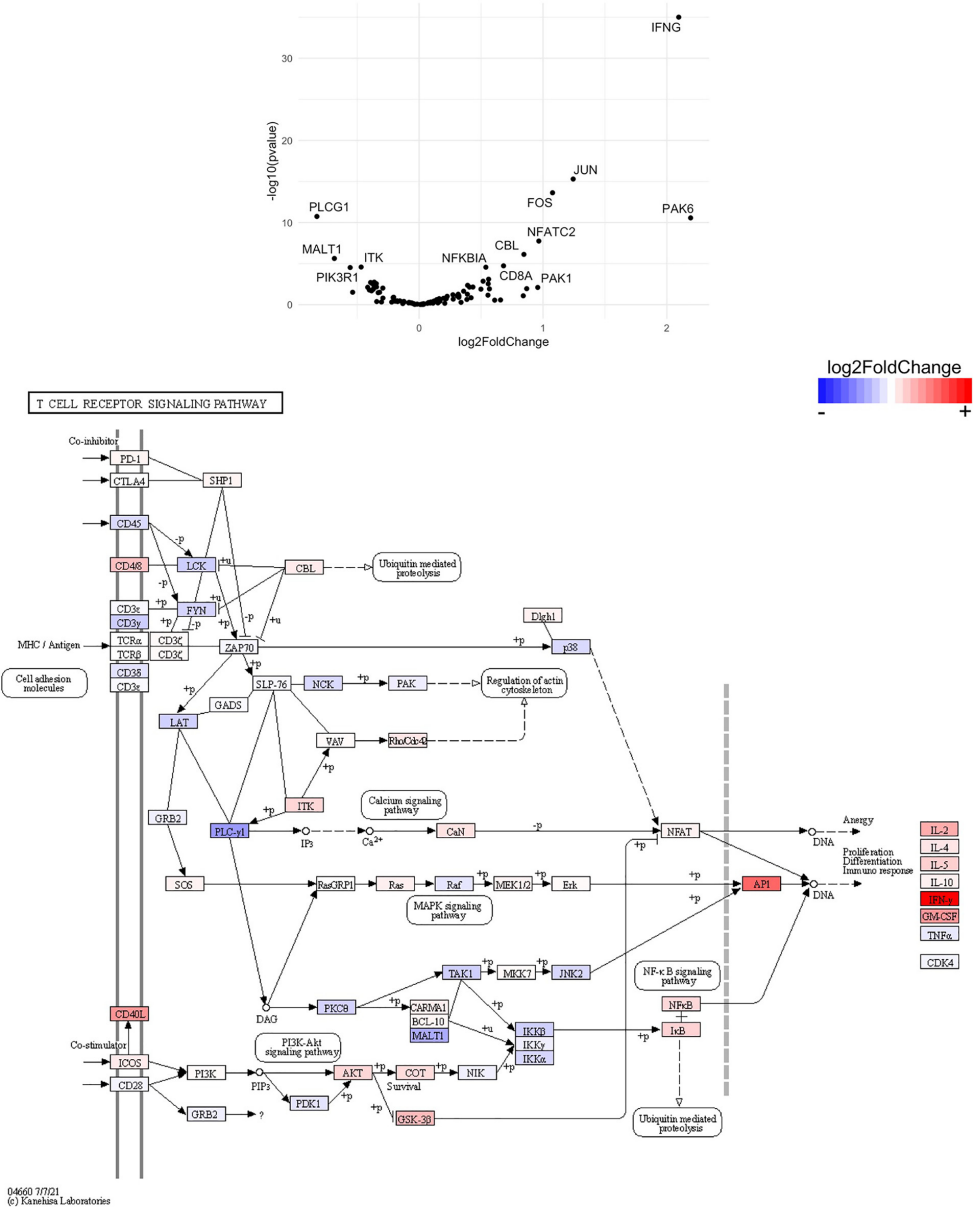
Genes related to T cell receptor signaling are differentially expressed in CD4^+^ T cells from the CNS of microglia-deficient B6J mice compared to microglia-deficient SJL mice. C57BL/6J and SJL/J mice were treated with a PLX5622-supplemented diet seven days prior to i.c. TMEV infection and sacrificed at 6 DPI for analysis (n = 10 mice per group). Volcano plot of RNA-seq transcriptome data and KEGG map display T cell receptor signaling-related gene expression in enriched CD4^+^ T cells from the CNS of microglia-deficient C57BL/6J mice relative to microglia-deficient SJL/J mice.

**Figure 15:**
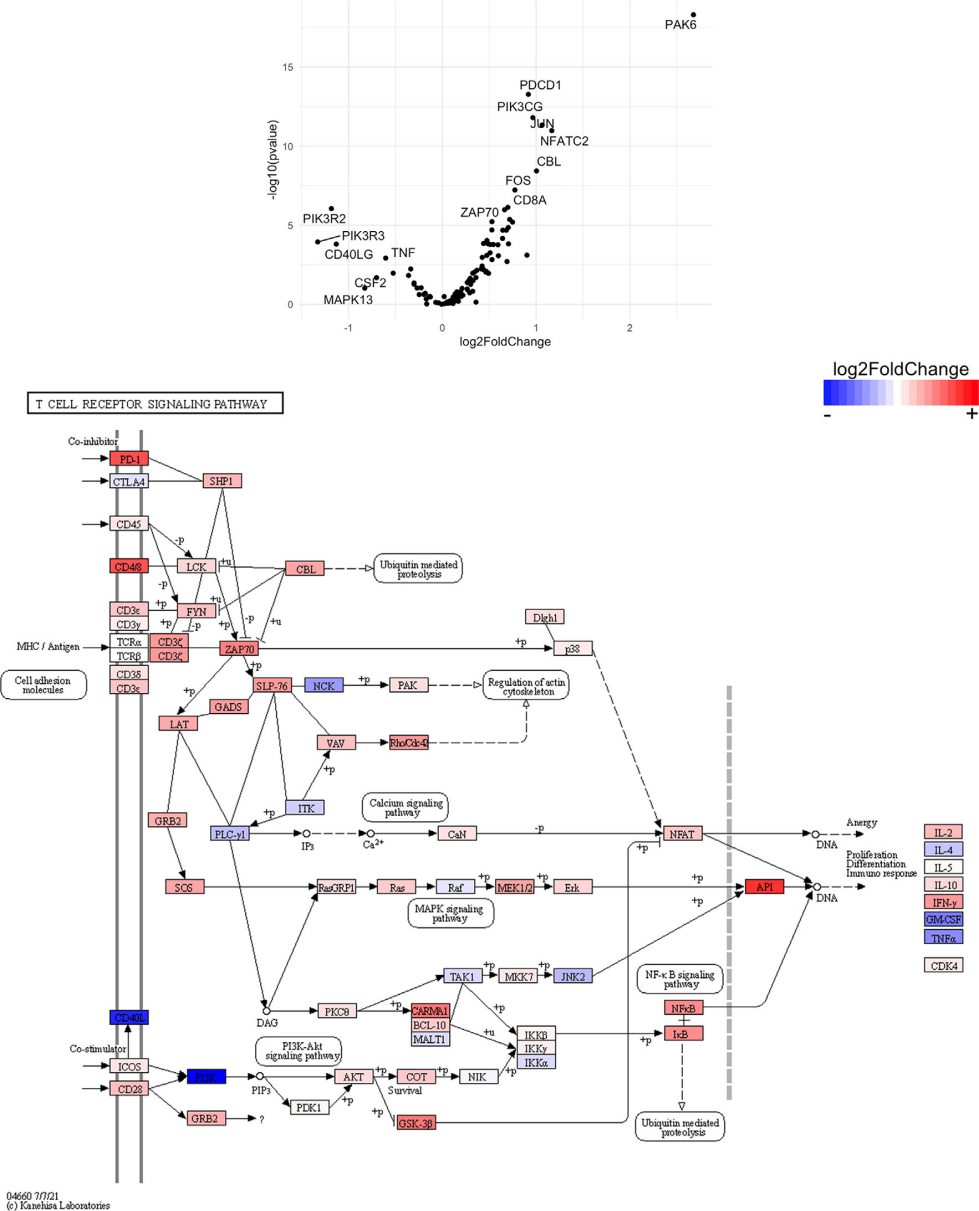
Genes related to T cell receptor signaling are enriched in CD8^+^ T cells from the CNS of microglia-deficient B6J mice compared to microglia-deficient SJL mice. C57BL/6J and SJL/J mice were treated with a PLX5622-supplemented diet seven days prior to i.c. TMEV infection and sacrificed at 6 DPI for analysis (n = 10 mice per group). Volcano plot of RNA-seq transcriptome data, censoring CD4 as an outlier that may represent contamination in CD8^+^ T cell enrichment (see **Supplemental Data**), and KEGG map display T cell receptor signaling-related gene expression in enriched CD8^+^ T cells from the CNS of microglia-deficient C57BL/6J mice relative to microglia-deficient SJL/J mice.

Interestingly, when we compare the transcriptome of CNS CD4^+^ and CD8^+^ T cells between microglia-deficient B6J and SJL mice, we observe enrichment in cytokine-cytokine receptor interaction and JAK/STAT signaling pathways in microglia-deficient B6J mice (**Figures 10–13** and **Supplemental Tables 13–16**). We do not measure enrichment in the T cell receptor signaling pathway in CNS CD4^+^ T cells comparing microglia-deficient B6J and microglia-deficient SJL mice (**Figure 14** and **Supplemental Table 17**), however we do measure enrichment in the T cell receptor signaling pathway in CNS CD8^+^ T cells in microglia-deficient B6J mice (**Figure 15**). Of note, CD4 is among the most upregulated genes in CNS CD8^+^ T cells of microglia-deficient SJL mice compared to microglia-deficient B6J mice, which may represent contamination during CD8^+^ T cell enrichment (**Figure 15** and **Supplemental Tables 18**). Additionally, chemokines/chemokine receptors (CCR1, CCL2, CCR2, CCL3, CCR8, CXCR5, CXCL10, CXCL11, CXCL12, CXCL13, CX3CR1, CX3CL1) and receptors (CSF1R) that are predominantly expressed in myeloid cells are also among the most differentially expressed genes comparing CNS CD4^+^ and CD8^+^ T cells within mouse strains (**Figures 8** and **9** and **Supplemental Tables 1–12**), suggesting contaminating myeloid gene expression detected by RNA-Seq. However, when we compare the transcriptome of CNS CD4^+^ and CD8^+^ T cells between microglia-competent B6J and SJL mice, we recapitulate previous findings regarding the response to TMEV in these strains including increased interferon (IFN) *γ* expression in B6J mice compared to SJL mice and increased interleukin (IL)-10 expression in SJL mice compared to B6J mice (**Supplemental Tables 19** and **20**) (31–33). Pathway analysis of CNS CD4^+^ and CD8^+^ T cell transcriptomes between mouse strains emphasizes that microglia are necessary to acutely limit effector immune pathways more so in B6J mice than SJL mice.

## Discussion

Microglia have been shown to be protective in the context of CNS infection with a variety of neurotropic viruses including picornavirus, flavivirus, coronavirus, rhabdovirus, and herpesvirus (11, 18, 19, 24, 25, 28, 34, 35). Here, we report that microglia play differential roles in neurotropic picornavirus infection depending on the host strain. In B6J mice, which normally develop acute behavioral seizures, but go on to clear TMEV, we and others have previously shown that microglia are critical for the antiviral response as microglia-deficient B6J mice succumb to fatal TMEV encephalitis (11, 24). This work identifies innate immune deficits and a deregulated T cell response as major contributors to the microglia-deficient B6J phenotype. Conversely, SJL mice, which are normally persistently infected with TMEV and develop a progressive demyelinating disease, do not succumb to acute fatal viral encephalitis when microglia are depleted. Instead, microglia-deficient SJL mice develop exacerbated TMEV-IDD associated with less T cell infiltration and lower T cell activation. This phenotype is partially reversible with repopulation of the microglia niche and partially recapitulated by depleting microglia at the onset of demyelinating disease. These host-dependent contributions of microglia in response to the same viral challenge highlight the complexity of the antiviral immune response in the CNS.

Acutely, we observe alterations in the innate response against TMEV in microglia-deficient B6J mice that are not present in microglia-deficient SJL mice. Importantly, the proportions of CNS monocytes/macrophages and lymphocytes were unaffected by microglia depletion at this acute time point (**Figures 1C, D**), in line with previous reports (19, 25, 26). Indeed, astrocytes have been shown to be a major producer of chemokines in the CNS in response to TMEV infection (36). We have previously shown that microglia-deficient B6J mice infected with TMEV exhibit demyelination, axon damage, and accumulation of TMEV antigen in the CNS, but only demyelination correlates with the titer of TMEV inoculum (11). In the current work, we find that the remaining microglia in microglia-deficient B6J mice have less MHC-II expression (**Figure 2D**). In agreement with our own findings, CNS infection of microglia-deficient B6J mice with the JHM strain of mouse hepatitis virus (JHMV), a neurotropic coronavirus, is associated with a similar decrease in MHC-II expression (18, 19). Microglia appear to be relatively more important than infiltrating monocytes/macrophages in the CNS antiviral response to TMEV as monocytes/macrophages in microglia-deficient B6J mice maintain MHC-II expression, but do not compensate for the loss of microglia. We do not distinguish between peripheral macrophages and CNS-resident macrophages including perivascular, meningeal, and choroid plexus macrophages in this work. However, we have previously shown that infiltrating peripheral macrophages comprise the vast majority of CNS macrophages in response to TMEV infection and that treatment with clodronate, which depletes both CNS-resident and peripheral macrophages, in B6J mice ameliorates the development of seizures in response to CNS TMEV infection while retaining the ability to clear virus (8, 37, 38). Therefore, while macrophages are dispensable for TMEV immunity in B6J mice, microglia are critical orchestrators of the antiviral immune response.

Interestingly, microglia depletion leads to an aberrant acute adaptive immune response to TMEV in B6J mice, but not in SJL mice. Microglia-deficient B6J mice infected with TMEV have less activated CD4^+^ helper T cells than microglia-competent B6J mice (**Figure 2H**), similar to what is observed with JHMV (18, 19). Despite lower expression of the activation marker CD25, *via* RNA-Seq we identify enrichment in genes related to cytokine-cytokine receptor interaction, JAK/STAT signaling, and T cell receptor signaling in CD4^+^ and CD8^+^ T cell populations from the CNS of microglia-deficient B6J mice compared to microglia-competent B6J mice (**Figure 8** and **Supplemental Tables 1–6**). Additionally, although TMEV-infected microglia-deficient B6J mice exhibit a decreased proportion of Tregs and a higher proportion of cytotoxic CD8^+^ T cells in the CNS (**Figures 2I, J**), they are hindered in their ability to clear TMEV (**Figure 2B**). The inability of microglia-deficient B6J mice to clear TMEV despite an upregulated proinflammatory T cell response is due in part to fewer microglia to present antigen and B6J-specific downregulation of antigen presentation machinery in microglia that remain. Microglia have been shown to induce Tregs *via* an MHC-II-dependent mechanism (39), thus the overall decreased capacity for antigen presentation in the CNS of B6J mice treated with PLX5622 contributes to a failure to regulate the acute TMEV immune response. Still other immunological functions of microglia, including phagocytosis, co-stimulation, and cytokine production, have been shown to be important to the antiviral response to other neurotropic viruses and we cannot rule out the importance of these functions in TMEV infection (3, 18, 27, 28).

SJL mice, in contrast to B6J mice, do not have the same acute deficiencies in the adaptive immune response in the context of reduced microglia and exhibit similar ability to clear TMEV compared to microglia-competent SJL mice at the acute timepoint (**Figure 2C**). This may be due to already low activation of CD4^+^ T cells in microglia-competent SJL mice compared to microglia-competent B6J mice (**Figure 2H**). Indeed, microglia-deficient SJL mice exhibit less enrichment in genes of the cytokine-cytokine receptor interaction, JAK/STAT, and T cell receptor signaling pathways among CD4^+^ and CD8^+^ CNS T cells compared to microglia-competent SJL mice (**Figure 9** and **Supplemental Tables 7–12**). Ultimately, while microglia-deficient B6J mice succumb to acute CNS TMEV infection, microglia-deficient SJL mice survive beyond the acute encephalitic phase of disease and well into the chronic demyelinating phase of disease (**Figure 3C**). Because different TMEV titers were used to infect B6J and SJL mice, each in line with titers previously used in these respective models, we cannot completely rule out the effect of inoculum amount on the difference we observe between mouse strains. Still, the enhanced mortality and pro-inflammatory response to a lower TMEV infectious dose in B6J mice compared to SJL mice suggests that the differences we observe are driven predominantly by host-intrinsic factors as opposed to differences in inoculum. Specifically, we find that IL-6, which we have previously shown to contribute to the development of seizures in B6J mice (40), and IL-17, which in combination with IL-6 has been shown to promote TMEV persistence (41), are upregulated in microglia-deficient B6J mice compared to microglia-deficient SJL mice (**Figures 10**, **11** and **Supplemental Tables 13** and **14**). Interestingly, the mechanism by which IL-6 and IL-17 promote TMEV persistence is through prevention of apoptosis and, indeed, anti-apoptotic molecules Bcl-XL and PIM1 are upregulated in microglia-deficient B6J mice compared to microglia-deficient SJL mice (**Figures 12** and **13** and **Supplemental Tables 15** and **16**). Additionally, we find that IL-15, which promotes T helper 1 cell responses (42), is also upregulated in microglia-deficient B6J mice compared to microglia-deficient SJL mice and is associated with increased IFN*γ* expression in CD4^+^ T cells from microglia-deficient B6J mice (**Figures 10**, **11** and **Supplemental Tables 13** and **14**). In terms of T cell receptor signaling, we find that the transcription factors nuclear factor (NF)-κB, nuclear factor of activated T cells (NFAT), and activator protein-1 (AP-1) that are important for activated T cell gene expression (43, 44) are upregulated in microglia-deficient B6J mice compared to microglia-deficient SJL mice (**Figures 14** and **15** and **Supplemental Tables 17** and **18**). Our disparate findings regarding the role of microglia in the B6J and SJL background suggest that microglia are necessary to suppress the robust inflammatory response that normally clears the virus in B6J mice whereas microglia are not critical to coordinate the less robust acute immune response that fails to clear TMEV in SJL mice.

While we did not observe a clear deficit in microglia-deficient SJL mice during acute TMEV infection, SJL mice chronically treated with PLX5622 exhibited more severe demyelinating disease (**Figure 3D**). This result is in accordance with studies of microglia depletion in JHMV, another model of virus-induced demyelination (18, 19, 26). However, we found that long-term PLX5622 treatment not only resulted in a decrease in the microglia population (**Figure 3B**), but also hindered the accumulation of T cells in the CNS (**Figure 4A**) and was associated with increased TMEV antigen in the spinal cord (**Figure 3G**). We also observed a decrease in the number of macrophages and T cells in the spleen with chronic PLX5622 treatment (**Figures 4F–H**). PLX5622 has previously been reported to affect myeloid and lymphoid cells in bone marrow, spleen and blood, though this likely depends on the duration of CSF1R antagonism and context of viral infection (28, 29, 34). Thus, the exacerbated demyelination and resulting paralysis in the context of CSF1R inhibition predominantly reflects a TMEV-mediated effect from failure to control viral replication as opposed to immunopathology.

To understand when CSF1R signaling is important in the CNS antiviral response to sustained TMEV infection, we inhibited CSF1R at different distinct phases of infection in SJL mice. Initiating PLX5622 during the acute phase of infection and cessation of PLX5622 at the onset of TMEV-IDD symptoms resulted in a reduction in mortality, weight loss, and TMEV antigen in the spinal cord compared to mice treated with long-term PLX5622 (**Figures 5D, E, G**). While clinical scores between animals switched off PLX5622 and animals maintained on PLX5622 were similar (**Figure 5C**), there was a trending decrease in clinical score in animals switched off PLX5622 that might have been more pronounced with additional time off CSF1R inhibition. Initiating PLX5622 at the onset of TMEV-IDD symptoms resulted in exacerbated clinical disease compared to microglia-competent mice (**Figure 7D**), though the difference with PLX5622 treatment was evident at a later timepoint (144 DPI) compared to when PLX5622 treatment was initiated prior to TMEV infection (103 DPI; **Figure 3D**). Interestingly, cessation of PLX5622 at the onset of TMEV-IDD increased the number of CNS T cells, but had similar helper T cell CD25 expression compared to long-term PLX5622 treatment (**Figures 6A, D**). In contrast, PLX5622 treatment at the onset of TMEV-IDD led to similar numbers of T cells in the CNS compared to mice with intact CSF1R signaling, however the helper T cells of PLX5622-treated mice were less activated as measured by CD25 expression (**Figures 7G, J**). Combined, these data suggest that at the chronic demyelinating phase of TMEV infection, CSF1R signaling is sufficient, but not necessary for CNS T cell accumulation and necessary, but not sufficient for T cell activation. In addition to T cell crosstalk, microglia also play a critical role in regeneration in the CNS. JHMV infection of microglia-deficient mice is associated with decreased spinal cord remyelination and lower expression of remyelination-associated factors in the spinal cord (18, 26). Therefore, the exacerbated clinical disease we observe with microglia depletion at the onset of TMEV-IDD may also be due to deficient microglia-derived remyelination and warrants further investigation.

B6J mice and SJL mice have been useful models to study differences in TMEV susceptibility. Genetically, the *H2d* locus has been shown to be a major genetic loci related to susceptibility to TMEV (45). Furthermore, the greater accumulation of Tregs in the CNS of SJL mice compared to B6J mice has been proposed to inhibit effector T function explaining, in part, why SJL mice are unable to clear TMEV and become persistently infected (46). We do not observe an increase in the proportion of CNS Tregs in microglia-competent SJL mice at the acute time point, however we do see less proinflammatory cytokine production and more anti-inflammatory cytokine production in CNS CD4^+^ helper T cells of SJL mice compared to B6J mice (**Supplemental Tables 19** and **20**). In addition to differences in helper T cell response, we find that the microglia response to TMEV infection is also critically different between SJL and B6J. Previous work has shown that microglia from B6J mice have higher expression of co-stimulatory molecules compared to microglia from SJL mice during TMEV infection (23). We add that SJL mice have a decreased microglial expression of MHC-II in acute TMEV infection compared to B6J mice (**Figure 2D**). This suggests that SJL mice develop a more tepid antiviral response compared to B6J mice, in part, due to decreased microglia-derived antigen presentation and co-stimulation.

In summary, this work provides evidence that the CSF1R-microglia axis is a critical orchestrator of the acute antiviral response to TMEV in B6J mice whereas the axis is more important in chronic stage of infection in SJL mice. Acutely, the protective effect of CSF1R signaling appears to be unique to microglia and not monocytes/macrophages, given that the infiltration of monocytes/macrophages into the CNS is intact in microglia-deficient B6J mice, but not sufficient to compensate for the loss of microglia. Antigen presentation *via* MHC-II is impeded in microglia-deficient B6J mice in microglia and is substantiated by an overactive T cell response that fails to control TMEV propagation. Chronic administration of the CSF1R inhibitor PLX5622 in SJL mice leads to exacerbated TMEV-IDD, however we observe reductions in not only CNS microglia, but also CNS T cells and splenic macrophages and T cells. This immunosuppression is reversible by discontinuing PLX5622 administration at the onset of disease and ameliorates TMEV-IDD, suggesting that CSF1R signaling is necessary for a protective antiviral response during sustained TMEV infection. Additionally, we observe exacerbated disease initiating PLX5622 at the onset of TMEV-IDD symptoms that is not associated with a decrease in the number of CNS T cells, but instead lower activation of CNS helper T cells. Thus, CSF1R signaling is important in the chronic phase of sustained TMEV infection to prime and activate a productive T cell response. These strain-dependent effects of the CSF1R-microglia axis in response to neurotropic picornavirus infection emphasize the diversity of the CNS innate immune response among closely related mammals as well as the protective proinflammatory and regulatory functions of microglia that determine susceptibility to CNS viral infection.

## Data Availability Statement

The datasets presented in this study can be found in online repositories. The names of the repository/repositories and accession number(s) can be found below: NCBI GEO, GSE160660.

## Ethics Statement

All animal studies were reviewed and approved by the University of Utah Institutional Animal Care and Use Committee and conducted in accordance with the guidelines prepared by the Committee on Care and Use of Laboratory Animals, Institute of Laboratory Animals Resources, National Research Council.

## Author Contributions

JS – idea generation, drafting and manuscript preparation, data collection, data analysis, and manuscript editing. AD-S – idea generation, data collection, data analysis, and manuscript editing DD – data collection. TH – data collection and data analysis. AT – data analysis. JL – manuscript editing. RF – idea generation and manuscript editing. All authors contributed to the article and approved the submitted version.

## Funding

This work was supported by the National Institutes of Health 5R01NS065714 (RF), 1R01NS065714 (RF), and 5T32AI055434 (JS).

## Conflict of Interest

The authors declare that the research was conducted in the absence of any commercial or financial relationships that could be construed as a potential conflict of interest.

## Publisher’s Note

All claims expressed in this article are solely those of the authors and do not necessarily represent those of their affiliated organizations, or those of the publisher, the editors and the reviewers. Any product that may be evaluated in this article, or claim that may be made by its manufacturer, is not guaranteed or endorsed by the publisher.
